# *Acanthamoeba* spp. in Contact Lenses from Healthy Individuals from Madrid, Spain

**DOI:** 10.1371/journal.pone.0154246

**Published:** 2016-04-22

**Authors:** Thiago dos Santos Gomes, Angela Magnet, Fernando Izquierdo, Lucianna Vaccaro, Fernando Redondo, Sara Bueno, Maria Luisa Sánchez, Santiago Angulo, Soledad Fenoy, Carolina Hurtado, Carmen del Aguila

**Affiliations:** 1 Facultad de Farmacia, Universidad San Pablo CEU, Urbanización Montepríncipe, Alcorcón, Madrid, Spain; 2 CAPES Foundation, Ministry of Education of Brazil, Brasília, DF 70040–020, Brazil; University of Birmingham, UNITED KINGDOM

## Abstract

**Purpose:**

*Acanthamoeba* keratitis (AK) is a painful and potentially blinding corneal infection caused by *Acanthamoeba* spp. In Madrid, environmental studies have demonstrated a high presence of these free-living amoebae in tap water. Since most of AK cases occur in contact lenses (CL) wearers with inadequate hygiene habits, the presence of *Acanthamoeba* in discarded CL has been studied and compared with other common etiological agents of keratitis, such as *Pseudomonas aeruginosa* and *Staphylococcus aureus*.

**Methods:**

One hundred and seventy-seven healthy individuals from Madrid contributed their discarded CL and answered a questionnaire on hygiene habits. DNA was extracted from the CL solution and analyzed by real-time PCR for *Acanthamoeba*, *Pseudomonas aeruginosa* and *Staphylococcus aureus*. These CL and their solutions were also cultured on non-nutrient agar to isolate *Acanthamoeba*.

**Results:**

Among the 177 samples, *Acanthamoeba* DNA was detected in 87 (49.2%), *P*. *aeruginosa* DNA in 14 (7.9%) and *S*. *aureus* DNA in 19 (10.7%). Cultivable amoebae, however, were observed in only one sample (0.6%). This isolate was genotyped as T4. The habits reported by this CL owner included some recognized risk factors for AK, but in this study only the practice of “not cleaning the CL case” presented some statistical significant association with *Acanthamoeba* DNA presence. Detection of the investigated bacterial DNA did not demonstrate statistical significant association with the studied practices, but the presence of *P*. *aeruginosa* revealed a possible inhibition of *Acanthamoeba* in these samples.

**Conclusions:**

The PCR results suggest a high presence of *Acanthamoeba* spp. in healthy CL wearers from Madrid, but we can assume that CL solutions are properly disinfecting the CL since only 1.1% of the positive PCR samples correspond to viable amoebae and, after four years, only one participant reported stronger ocular problems. Nevertheless, more studies are necessary to corroborate this hypothesis.

## Introduction

The free-living amoebae *Acanthamoeba* are ubiquitous in nature and have been isolated from soil, dust, air, seawater, swimming pools, sewage, sediments, air-conditioning units, domestic tap water, bottled water, dental treatment units, hospitals, dialysis apparatus, eyewash stations, contact lenses as well as their lens cases and as contaminants in bacterial, yeast and mammalian cell cultures [1, 2].

This genus comprises more than 21 species, a classification that has lately been shown to be inconsistent because correlation between binomial classification and molecular typing is, in most cases, not concordant. For this reason, a classification of *Acanthamoeba* based on the 18S ribosomal RNA full gene sequence has been proposed [3]. Following this classification, 20 genotypes for *Acanthamoeba* have so far been described [4, 5]. With regard to the distribution of these *Acanthamoeba* genotypes, studies in environmental and clinical samples have shown T4 to be the most common genotype either in the environment or as the causative agent of various diseases [6].

The ability to produce serious human and animal infections has attracted attention to these protozoa lately [7, 8]. *Acanthamoeba* keratitis (AK), one of these infections, is a painful and severe sight-threatening ulceration of the cornea, in which the lesions can cause extensive ocular damage. If the infection is not diagnosed early and aggressively treated, enucleation may be required [9, 10]. The number of AK cases diagnosed has increased dramatically over the last 20 years, and over 3000 cases have been estimated to occur in the United States alone [11]. Contact lens (CL) wearers are at risk of acquiring AK or microbial keratitis caused by different bacterial agents, mainly *Pseudomonas aeruginosa* and *Staphylococcus aureus*. CL wearers with poor hygiene practices, one of the recognized risk factors, as well as failure to comply with the recommended cleaning and disinfection procedures, rinsing with tap water or homemade saline solutions, showering while wearing lenses and the overuse of disposable CL are at higher risk for eye infections [12–15].

The relation between CL wearers and AK has been demonstrated by several authors, leading to increased attention on CL and their containers. Variable contamination rates have been demonstrated in CL or CL cases according to the different geographic regions. Most studies were conducted using culture methods, with the exception of a research group from Hong Kong that analyzed CL cases through conventional PCR, demonstrating a contamination rate of 1% [16]. In other countries, studies conducted using culture methods demonstrated contamination rates such as 6.9% in the United Kingdom [17], 8% in New Zealand [18], 4.2 to 15.1% in regions of Korea [19, 20], 9% in the southern region of Brazil [21], 10% in Iran [22] and a surprising 65.9% in the Canary Islands, Spain [8].

In Spain, *Acanthamoeba* keratitis cases have also been found [23–33]. As in other Mediterranean countries, most amoebic isolates from humans belonged to the T4 genotype, although occasionally other *Acanthamoeba* genotypes have been diagnosed [24, 34]. The especially high presence of *Acanthamoeba* spp. in CL cases from the Canary Islands presents disturbing data [8]. However, there is little information concerning this infection in the Spanish population.

Environmental studies on *Acanthamoeba* epidemiology in the Canary Islands (Spain) demonstrated that amoebae concentrations for this area were as high as 59% in tap water and 40% in sea water [35]. Thus, the high contamination rate exhibited in CL cases from the Canary Islands may be explained by the high presence of this amoeba in the environment. In Central Spain, there are only four reports of *Acanthamoeba* spp. identification. The first report demonstrated the presence of this amoeba in drinking water fountains while the other reports investigated the presence of *Acanthamoeba* spp. in raw and finished water from drinking water treatment plants (DWTP) and wastewater treatment plants (WWTP), indicating not only the presence of these amoebae but also one of the highest rates of such amoebae in water samples reported to date. More importantly, *Acanthamoeba* spp. was detected in the finished water of all DWTPs, indicating that the purification processes used in these treatment plants did not eliminate these protozoans [31, 36–38]. Thus, the high presence of *Acanthamoeba* spp. in tap water from Madrid led us to investigate if exposure to this high environmental presence is associated to CL contamination and the possible additional factors that could increase the risk of AK acquisition by CL wearers of this area.

## Materials and Methods

### CL Samples, Investigated Habits and Follow-up

Between October 2011 and September 2012, discarded CL and their cases were donated by 177 healthy individuals from Madrid, who also answered a hygiene habits questionnaire at the time of their CL collection by the laboratory. The habits investigated in this study were: CL overuse, not washing hands before handling CL, rinsing CL with tap water, sporadically or usually showering while using lenses, no daily change of CL case solutions and not cleaning the CL cases. Participants agreed to collaborate in this study by signing a written form attesting their free and informed consent, in accordance with Constitutional Act 15/1999 and Royal Decree 1720/2007, of Personal Data Protection, and Law 14/1986, General Health Law, from Spain. These laws define that access to the clinical record for judicial, epidemiological, public health, research or educational purposes carry an obligation to keep the patient’s personal identification data separated from clinical and healthcare data, so that, as a general rule, anonymity is ensured. Additionally, this study was reviewed and approved by the Research Ethics Committee of the Universidad San Pablo-CEU, with protocol number 109–15. In this form, the participants also indicated their wish to be informed about positive results and, if so, an e-mail address was given for this purpose. After approximately four years of their participation, CL owners with positive results were contacted through those e-mail addresses and questioned about their eye health during this period.

### Molecular Methods

#### DNA extraction

DNA was extracted from approximately 200 μl of CL solutions concentrated by centrifugation (1500 rpm/10 min) by using the DNAeasy® Blood & Tissue kit (QIAGEN, Valencia, CA, USA) following the manufacturer’s instructions.

#### Real-time PCR assay for *Acanthamoeba* DNA detection

A TaqMan real-time PCR for the detection of *Acanthamoeba* was performed to test the DNA samples extracted from CL solutions, as well as positive and negative controls, following a protocol previously described in the literature [11]. The positive control used in the real-time PCR was genomic DNA from the *Acanthamoeba* USP-CR5-A35 (genotype T4).

#### Real-time PCR assay for DNA detection of *Pseudomonas aeruginosa* and *Staphylococcus aureus*

A multiplex protocol of TaqMan real-time PCR for the detection of these two bacterial species was performed to test the DNA samples extracted from CL solutions, as well as positive and negative controls. The assay was adapted from a protocol previously described by Gadsby *et al* [39]. In this study, a multiplex real-time assay was conducted with the same cycling conditions of the original protocol, coupling in the same reaction the specific primers and probes designed for *Pseudomonas aeruginosa* and *Staphylococcus aureus*, which were the desired target in this study.

#### Sequencing

Primers JDP1 and JDP2, which amplify a fragment of approximately 500 bp of the ASA.S1 region of the 18S RNA gene, were used for *Acanthamoeba* genotyping of isolated amoebae as described previously [40]. PCR amplicons were purified with NucleoSpin Extract II (Macherey-Nagel, Düren, Germany) following the manufacturer’s instructions and sequenced at both ends with PCR primers by Macrogen laboratories sequencing service (Seoul, Korea). The sequences analysis was done with Bioedit Sequence Alignment Editor 7.0.5.3 [41]. To determine the genotypes, sequencing data was aligned with *Acanthamoeba* genotype sequences available in the GenBank database.

### *Acanthamoeba* culture

Contact lenses and approximately 80 μl of the concentrated CL solution were inoculated onto 2% Neff’s saline non-nutrient agar plates seeded with heat-killed *Escherichia coli* and incubated at 28°C. The cultures were monitored daily for 20 days. Trophozoites or cysts observed were subcultured by transferring small pieces of agar containing amoebae to a fresh plate in order to isolate it from other microrganisms. Axenization of the cultures was achieved by transferring small pieces of agar to PYG medium (0.75% proteose peptone, 0.75% yeast extract, and 1.5% glucose with 40 μg gentamicin per milliliter) at 28°C without shaking. Isolated amoebae were submitted subsequently to a sequencing procedure to determine the genotype.

### Statistical Analysis

This analysis was conducted considering the (bilateral) asymptotic significance obtained by Pearson’s chi-square test (p < 0.05), using the SPSS Statistics v20 software (IBM Corporation, Armonk, NY, USA).

## Results

One hundred seventy-seven CL users contributed to this study donating their discarded lenses and answering a questionnaire about their hygiene practices and precautions concerning the use of their lenses. However, 9 individuals failed to return their completed questionnaires and the comparative analysis of these practices was conducted for only 168 individuals. Their ages varied between 11 and 83, with a mean age of 29.7 years. Additional characteristics reported about the donated lenses are shown in Table 1.

**Table 1:** Main characteristics of the donated contact lenses (CL) and their users (n = 168).

Main characteristics		Percentage
**CL User Gender**	*Female*	70.2%
	*Male*	29.8%
**CL Type**	*Soft*	98.2%
	*Hard*	1.8%
**Frequency of Use**	*Daily*	87.5%
	*Occasionally*	12.5%
**Produces eye discomfort**	*Yes*	53.6%
	*No*	46.4%

Regarding the practices reported by volunteers in the survey, we noted that a large percentage of them performs some of the behaviors considered as risk factors for *Acanthamoeba* spp. infection regularly or sporadically. For example, overuse and the habit of sporadically showering using CL were reported by over 40% of volunteers.

In addition, in order to investigate a possible association between the report of these practices and presence of *Acanthamoeba*, a real-time PCR was performed, detecting the DNA of *Acanthamoeba* in 87 of 177 samples (49.2%). These results distributed by gender also revealed a very similar frequency of positive samples in male (52%) and female participants (49.2%), exhibiting an odds ratio of contamination of male CL wearers compared to female of 1.11. However, the statistical analysis of the possible association between the report of these practices and the detection of amoebic DNA by real-time PCR in the studied samples did not find an associative effect with statistical significance for most of the investigated habits. The only practice that demonstrated some association with this infection was the habit of not cleaning the lens cases, a behavior reported by 29.2% of the CL wearers participating in this study. According to Pearson’s chi-square (χ²) test, it can be determined with 94% confidence that this habit can be closely related with infection by these amoebae (p = 0.062).

Also, a real-time PCR was performed for the detection of the two most common species associated to microbial keratitis, *P*. *aeruginosa* and *S*. *aureus*, in order to evaluate possible association between the presence of *Acanthamoeba* and these bacteria. Regarding possible association between the hygiene habits and the detection of both species bacterial DNA, the analysis did not find an associative effect with statistical significance (S1 Table). The presence or absence of *S*. *aureus* DNA also did not demonstrate an effect on the presence of *Acanthamoeba*. However, the presence of *P*. *aeruginosa* demonstrated a possible effect on the presence of *Acanthamoeba* in these samples, revealing a higher amoebic presence in the absence of this bacteria. The comparative analysis between the detection of amoebic DNA and the principal reported practices or bacterial presence is shown on Table 2.

**Table 2:** Detection of *Acanthamoeba*’s DNA and the association with habits reported / bacterial presence.

Habits Reported / Bacterial presence	Total % of volunteers w/	PCR (+) % in individuals w/	PCR (+) % in individuals w/o	P-value^a^
CL overuse	42.8% (72/168)	45.8% (33/72)	53.1% (51/96)	0.350
Not washing hands before handling CL	20.8% (35/168)	54.3% (19/35)	48.9% (65/133)	0.569
Rinsing CL with tap water	22.0% (37/168)	40.5% (15/37)	52.7% (69/131)	0.193
Sporadically showering while wearing CL	41.1% (69/168)	49.3% (34/69)	48.8% (20/41)	0.948
Usually showering while wearing CL	34.5% (58/168)	51.7% (30/58)	48.8% (20/41)	0.948
No daily exchange of CL case solutions	32.1% (54/168)	48.1% (26/54)	50.9% (58/114)	0.741
Not cleaning the CL case	29.2% (49/168)	61.2% (30/49)	45.4% (54/119)	0.062
*Pseudomonas aeruginosa*	7.9% (14/177)	21.4% (3/14)	51.5% (84/163)	0.031
*Staphylococcus aureus*	10.7% (19/177)	57.9% (11/19)	48.1% (76/158)	0.420

PCR (+), positive result in PCR; w/, with; w/o, without.

^a^ P-value: Corresponds to the (bilateral) asymptotic significance obtained in Pearson's χ² test (p<0,05).

**Fig 1:**
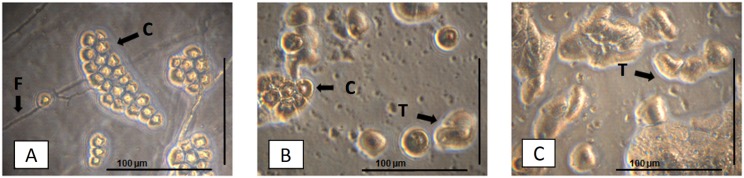
Photograph of amoebae isolated by culture from contact lenses. A) *Acanthamoeba* cysts and fungi isolated from the positive sample in non-nutrient agar. B) Cysts and trophozoites transferred to a second plate for their isolation. C) Isolated trophozoites. This figure was captured using the inverted microscope Eclipse TS100 (Nikon) coupled to the DS Camera Control Unit DS-L2 (Nikon). In the figure, structures are indicated as: fungi (F), cysts (C) and trophozoites (T).

These CL and their solutions were also cultured on non-nutrient agar plates seeded with inactivated *E*. *coli*. Nevertheless, the presence of cultivable amoebae was observed in only one sample, indicating a presence of only 0.6% by culture. This sample also presented DNA amplification on real-time PCR, demonstrating a consistent result between these 2 methods for this sample. This CL owner was a female who reported some habits considered as risk factors, such as extended use of CL, not washing hands before handling CL, eventually rinsing CL with tap water and showering while using these lenses. The isolation of these amoebae in culture can be observed in Fig 1.

These trophozoites were isolated and genotyped as T4/16, according to the classification previously established [42]. The genotype T4 is the most common type found worldwide and in *Acanthamoeba* infections. Also, data concerning molecular epidemiology of *Acanthamoeba* species are relevant since the type of isolate present in a keratitis patient may influence the outcome of the chemotherapy [43].

Furthermore, this CL owner reported feeling some eye discomfort, the same symptom reported by 53.6% of the participants. This symptom can indicate some pathology, such as AK or some other infection, caused by bacteria or fungi. Therefore, the eye discomfort complaint for each habit reported was analyzed as well as a possible association of the habits and eye discomfort with the presence of *Acanthamoeba* DNA. The results obtained are shown on Table 3.

**Table 3:** Report of eye discomfort for each habit or bacterial presence and the association with the presence of *Acanthamoeba* DNA.

Habits Reported / Bacterial presence	% of individuals w/ eye discomfort for each feature	PCR(+) % in individuals w/ eye discomfort for each feature	P-value^a^
CL overuse	56.9% (41/72)	51.2% (21/41)	0.857
Not washing hands before handling CL	51.4% (18/35)	61.1% (11/18)	0.318
Rinsing CL with tap water	64.9% (24/37)	37.5% (9/24)	0.186
Sporadically showering while wearing CL	60.9% (42/69)	52.4% (22/42)	0.722
Usually showering while wearing CL	46.5% (27/58)	59.3% (16/27)	0.294
No daily exchange of CL case solutions	55.5% (30/54)	43.3% (13/30)	0.420
Not cleaning the CL case	63.3% (31/49)	64.5% (20/31)	0.073
*Pseudomonas aeruginosa*	57.1% (8/14)	25.0% (2/8)	0.148
*Staphylococcus aureus*	36.8% (7/19)	57.1% (4/7)	0.700

PCR (+), positive result in PCR; w/, with; w/o, without.

^a^ P-value: Corresponds to the (bilateral) asymptotic significance obtained in Pearson's χ² test (p<0,05).

This analysis indicates a high incidence of eye discomfort for each habit investigated. However, this symptom does not always seem to be connected with a high detection rate for *Acanthamoeba* DNA in these individuals. In fact, the only habit in which the report of eye discomfort presents a slight tendency of association with amoebic DNA presence was the habit of not cleaning the lenses cases, as it was observed in the previous analysis. For the other practices, the high percentage of reported eye discomfort might be associated with the presence of other microorganisms, pathogenic or not.

At February 2016, an attempt of follow-up was conducted with the 87 participants that had a positive result for *Acanthamoeba*. Among these 87 participants, 42 individuals (48.3%) informed about their ocular health since they contributed to this study, 18 individuals (20.7%) chose not to be informed about the results and 27 (31.0%) did not return the attempt of contact. With the exception of one participant, none of the participants who answered our follow-up request suffered of keratitis in this period, reporting only viral conjunctivitis in a few individuals. Only one CL owner informed stronger ocular problems, reporting several conjunctivitis and recurrent keratitis in the last months. Specific diagnostic methods for the detection of *Acanthamoeba* were offered to this participant, but no other answer was received.

## Discussion

In recent decades, AK has been recognized as an emerging disease, especially after the occurrence of some recent outbreaks [43]. One risk factor in AK is the use of CL exposed to contaminated water, usually due to a lack of hygiene habits. Over 85% of AK cases occur in CL wearers and due to their increasing number, it is important to assess the possible associated risks, and to make both current and new users aware of these risks [7, 8].

In this study, *Acanthamoeba* culture revealed the presence of cultivable amoebae in only one sample (0.6%), a surprisingly low incidence if compared to the 65.9% encountered in the CL analyzed by the same method in the Canary Islands (Spain) and the high incidence (93.8%) of *Acanthamoeba* found in the finished waters from DWTP in Madrid. Environmental studies concerned with the epidemiology of *Acanthamoeba* in the Canary Islands (Spain) demonstrated a high presence of these amoebae in the area, which can be associated with the high contamination rate found in CL cases from this population. Thus, it would be expected to find similar results in Madrid, however, this did not occur. A possible reason for the large difference between theses 2 geographical regions could be related to the warm climate and dust found in the Canary Islands, very different from the climate of the central region of Spain [8, 31, 35].

However, the performed real-time PCR demonstrated a much larger incidence value in these samples, showing 49.2% of positive samples. This difference with the culture method could be explained as the culture of specimens is a highly specific diagnostic method, but also limited by high false-negative rates. Studies in the United States and United Kingdom concerning culture methods reported sensitivity values from 50 to 57% [44]. Moreover, large-volume specimens such as observed in contact lens solutions have a demonstrable dilutional effect and are therefore subject to poor smear and culture results. Thus, diagnostic methods such as PCR that detect very few organisms in a clinical specimen are clearly more sensible in samples where the burden of *Acanthamoebae* is low [45].

This molecular approach to *Acanthamoeba* spp. can reveal a higher contamination rate on CL and, thus, more CL wearers at risk of acquiring AK. However, until now this methodology has not been widely used for *Acanthamoeba* detection. In Honk Kong, a study using conventional PCR analysis, conducted on tap water, has revealed 10% of tap waters contaminated and 1% of contaminated CL cases [16]. In this case, the low percentage of contaminated CL cases may be due in part to the low presence of this amoeba in the environment but also to a lower detection using conventional PCR. In Madrid, an environmental study has indicated a presence of 100% by real-time PCR [31], so it is reasonable to expect a higher presence of *Acanthamoeba* on CL.

Furthermore, the real-time PCR assay selected for this study used PCR primers and TaqMan probes targeting regions of the nuclear small subunit ribosomal (18S rRNA) gene. This gene is a robust diagnostic target for PCR because it is part of a ribosomal repeat unit that is present in multiple copies in each cell. It has been estimated that *Acanthamoeba* has approximately 600 copies of this ribosomal unit. Upon disruption, each amoeba cell will thus release hundreds of target molecules, resulting in an assay being deemed to have high sensitivity [11].

These real-time PCR results are also uniformly distributed over the collection year of these samples and are consistent with the constant environmental presence of *Acanthamoeba* spp. demonstrated in Madrid [31, 37]. This association between contamination rate and environmental distribution has already been described for another region. A study in Iowa City (Iowa, USA) demonstrated two peak periods per year when the onset of AK symptoms was most frequent (June and November), corresponding closely with the seasonal concentration of amoebae found in Tulsa (Oklahoma, USA), a region with similar climatic conditions [46, 47].

Another factor that could be involved in the detection of only 1.1% of viable amoebae among samples presenting DNA amplification is the prolonged exposure time of these CL to their solutions before sample collection. The interval between the last use of the donated CL and their arrival at the laboratory for analysis is unknown. If it was a prolonged time, the amoebae could have perished due to lack of nutrients or by the supposed anti-*Acanthamoeba* effect of these solutions. This is because the exposure time to the different solutions against the amoebae is very important for the biocide effect. Some studies have already demonstrated a low efficiency of these solutions in exposure times of 4 to 6 hours, increasing with longer periods of incubation. However, most CL wearers do not leave their CL exposed to these solutions for more than 8 to 12 hours, which corresponds to the night period [48]. These differences of effectivity according to exposure time are important and can explain the greater detection by molecular methods than culture, since only viable protozoans can be detected by culture while molecular methods such as PCR can detect DNA, even when parasites are no longer alive.

Another real-time PCR assay was also performed to evaluate the presence of bacteria in these lenses, more specifically *P*. *aeruginosa* and *S*. *aureus*. These species are the most common bacterial agents associated to microbial keratitis [14, 15]. The comparative analysis between their presence and the presence of *Acanthamoeba* demonstrated no difference to *S*. *aureus* while the presence of *P*. *aeruginosa* seemed to be inhibiting the presence of this amoeba (p = 0.031). This datum converge to the information suggested in the scientific literature where it has already been speculated an inhibitory effect from this bacterium on amoebic growth and survival [49].

Regarding the obtained information on hygiene habits, no statistically significant association was observed between the reported habits and the presence of bacterial DNA. Meanwhile, among individuals participating in this study, the practice “not cleaning the CL case” indicated a possible association between this behavior and the presence of amoebic DNA, with statistical significance, i.e., individuals with this habit may have more chance of exposure to the parasite. This is an important datum to consider once this practice had been reported by approximately one third of the participants and may be a commonly disseminated habit among CL wearers. However, only 1.1% of the positive PCR samples in this study correspond to viable amoebae and, after four years, only one participant reported stronger ocular problems, so there is a possibility that commercial CL solutions are properly disinfecting these CL.

Nevertheless, more attention to the recommendation of cleaning the CL cases should be given and, more importantly, CL wearers should be informed of more detailed instructions about alternative possible cleaning regimens. The manufacturer’s current guidelines (rinse and air-dry) have already been evaluated in some studies and more effective cleaning regimens have already been described for the elimination of bacterial biofilms attached to CL cases interior surface. A more effective instruction of cleaning procedure consists in a four-step strategy: rub (with a clean finger and a fresh multipurpose disinfecting solution for 5 seconds), rinse (with a fresh solution), tissue-wipe and air-dry (face down on a face tissue for six hours) [50, 51]. Considering that amoebic trophozoites also tend to attach to surfaces, this recommendation is advisable to eliminate *Acanthamoeba* and prevent different types of keratitis, including AK. These frictional forces included in the CL case cleaning strategy might help removing the adhered trophozoites even when the biocide chemicals fail to eliminate the parasites.

Although other practices investigated did not present statistical significant values for their possible association with the presence of amoebic DNA, the same attention should be given to the risk these habits can offer. This is because the presence or absence of these habits could also be affected by the number of participants in this study or to less reporting of some practices due to inhibition or the embarrassment of some volunteers.

Finally, we must consider that these practices, investigated collectively in this study, had already been suggested separately in the scientific literature as additional risk factors for this infection, but their actual contribution had never before been evaluated. Even though we have found a possible association to only one of the investigated habits, it is general agreed that the use of contact lenses is a risk factor for *Acanthamoeba* infection. Thus, it is important to continue to assess whether or not these studied habits, or other practices, can influence the presence of the amoebae and act as additional risks factors to *Acanthamoeba* infection.

## Supporting Information

S1 Table Detection of *Pseudomonas aeruginosa* and/or *Staphylococcus aureus* and the association with habits reported. (DOCX)
