# Supplementary material for: Acanthamoeba spp. in Contact Lenses from Healthy Individuals from Madrid, Spain
Source: PLoS One. 2016 Apr 22;11(4):e0154246. doi: 10.1371/journal.pone.0154246 (PMC4841564; doi:10.1371/journal.pone.0154246)
Supplement: S1 Table — (DOCX) [file pone.0154246.s001.docx]

**S1 Table.** **Detection of *Pseudomonas aeruginosa* and/or *Staphylococcus aureus* and the association with habits reported.**

| **Habits Reported** | **PCR (+) % in individuals w/ these habits** | **PCR (+) % in individuals w/o these habits** | **P-value^a^** |
| --- | --- | --- | --- |
| ***Pseudomonas aeruginosa*** |  |  |  |
| CL overuse | 12.5% (9/72) | 5.2% (15/96) | 0.091 |
| Not washing hands before handling CL | 14.3% (5/35) | 6.8% (9/133) | 0.152 |
| Rinsing CL with tap water | 10.8% (4/37) | 7.6% (10/131) | 0.537 |
| Sporadically showering while wearing CL | 8.7% (6/69) | 12.2% (5/41) | 0.456 |
| Usually showering while wearing CL | 5.2% (3/58) | 12.2% (5/41) | 0.456 |
| No daily exchange of CL case solutions | 11.1% (6/54) | 7.0% (8/114) | 0.370 |
| Not cleaning the CL case | 6.1% (3/49) | 9.2% (11/119) | 0.506 |
|  |  |  |  |
| ***Staphylococcus aureus*** |  |  |  |
| CL overuse | 11.1% (8/72) | 11.5% (11/96) | 0.944 |
| Not washing hands before handling CL | 11.4% (4/35) | 11.3% (15/133) | 0.980 |
| Rinsing CL with tap water | 8.1% (3/37) | 12.2% (16/131) | 0.486 |
| Sporadically showering while wearing CL | 13.0% (9/69) | 12.2% (5/41) | 0.720 |
| Usually showering while wearing CL | 8.6% (5/58) | 12.2% (5/41) | 0.720 |
| No daily exchange of CL case solutions | 13.0% (7/54) | 10.5% (12/114) | 0.641 |
| Not cleaning the CL case | 14.3% (7/49) | 10.1% (12/119) | 0.434 |
|  |  |  |  |
| ***P. aeruginosa* and/or *S. aureus*** |  |  |  |
| CL overuse | 23.6% (17/72) | 15.6% (15/96) | 0.192 |
| Not washing hands before handling CL | 25.7% (9/35) | 17.3% (23/133) | 0.259 |
| Rinsing CL with tap water | 18.9% (7/37) | 19.1% (25/131) | 0.982 |
| Sporadically showering while wearing CL | 20.3% (14/69) | 24.4% (10/41) | 0.393 |
| Usually showering while wearing CL | 13.8% (8/58) | 24.4% (10/41) | 0.393 |
| No daily exchange of CL case solutions | 24.1% (13/54) | 16.7% (19/114) | 0.253 |
| Not cleaning the CL case | 18.4% (9/49) | 19.3% (23/119) | 0.885 |

PCR (+), positive result in PCR; w/, with; w/o, without.

^a^ P-value: Corresponds to the (bilateral) asymptotic significance obtained in Pearson's χ² test (p<0,05).
